# Moving the Plasmon of LaB_6_ from IR to Near-IR via Eu-Doping

**DOI:** 10.3390/ma11020226

**Published:** 2018-02-01

**Authors:** Tracy M. Mattox, D. Keith Coffman, Inwhan Roh, Christopher Sims, Jeffrey J. Urban

**Affiliations:** Molecular Foundry, Lawrence Berkeley National Laboratory, One Cyclotron Rd., Berkeley, CA 94720, USA; dcoffm5261@gatech.edu (D.K.C.); noinhwan@gmail.com (I.R.); christophersims2017@u.northwestern.edu (C.S.)

**Keywords:** plasmon, hexaboride, doping, lanthanum hexaboride, LaB_6_

## Abstract

Lanthanum hexaboride (LaB_6_) has become a material of intense interest in recent years due to its low work function, thermal stability and intriguing optical properties. LaB_6_ is also a semiconductor plasmonic material with the ability to support strong plasmon modes. Some of these modes uniquely stretch into the infrared, allowing the material to absorb around 1000 nm, which is of great interest to the window industry. It is well known that the plasmon of LaB_6_ can be tuned by controlling particle size and shape. In this work, we explore the options available to further tune the optical properties by describing how metal vacancies and Eu doping concentrations are additional knobs for tuning the absorbance from the near-IR to far-IR in La_1−x_Eu_x_B_6_ (x = 0, 0.2, 0.5, 0.8, and 1.0). We also report that there is a direct correlation between Eu concentration and metal vacancies within the Eu_1−x_La_x_B_6_.

## 1. Introduction

Plasmonic nanoparticles are well known for their intriguing properties [1], and are being explored in a variety of fields such as photovoltaics [2], nanosensors [3], drug delivery devices [4], and quantum optics [5]. The physical properties of plasmonic materials are typically easy to tune because of their high carrier concentration and small size, where seemingly minor adjustments such as altering the particle shape or size have a substantial influence on the absorbance spectrum [1]. Vacancies also play a large role in tuning the optical properties of such materials, having a significant influence on free carrier density and doping constraints [6,7]. It′s even possible to fully tune the plasmon independent of dopant concentration in core-shell indium-tin-oxide nanoparticles [8] and by reducing holes in the valence band in copper sulfide [9].

Plasmonic materials are highly sought after in the windows industry. The ability to design a material to selectively transmit in the visible region while absorbing the most intense radiative heat in the IR (about 750 nm–1250 nm) is important for smarter window design, especially in hot climates. [10,11,12] Metal hexaborides (MB_6_) are being sought after for these applications, and with lanthanum hexaboride (LaB_6_) absorbing in the middle of this range (~1000 nm) [13,14] we focus our efforts here on the tuning of LaB_6_. It has already been shown that changing the particle size of LaB_6_ nanoparticles offers a means of controlling the plasmon [15,16] and that these particles may be incorporated into polymers to make films [17,18]. Though some work has been done on LaB_6_ to study how La vacancies influence vibrational energies [19] and how doping impacts the thermionic power [20,21], there is a potential link between doping content and vacancies in LaB_6_ that has gone unexplored. Given the ability of doping levels and metal vacancies to alter free electron concentrations and thus the optical properties in Eu_1-x_La_x_B_6_, we wished to explore the possible connection between doping concentration and metal vacancies as an additional means of controlling the plasmon.

In this work we demonstrate the possibility of alloying LaB_6_ nanoparticles with Eu using, for the first time, a low temperature solid state technique with varying ratios of Eu to La. Interestingly, we report there is a direct correlation between Eu concentration and metal (M) vacancies within the Eu_1-x_La_x_B_6_ system. Furthermore, this method allows the plasmon to be tuned across an incredibly large absorbance range from 1100 nm to 2050 nm, which may open doors to new optoelectronic applications.

## 2. Experimental Procedures

Anhydrous lanthanum (III) chloride (99.9% pure, Strem Chemical), anhydrous europium (III) chloride (99.99% pure, Strem Chemical) and sodium borohydride (EMD) were used as received and stored in an argon atmosphere glove box until use. Reactant powders were a stoichiometric 6:1 ratio of NaBH_4_ to metal chloride, where the metal chloride content was a mixture of EuCl_3_ and LaCl_3_ with varying ratios of (Eu:La). The mixtures were transferred to alumina boats approximately two inches long and 1 cm wide and the reactions run in a one-inch diameter quartz tube in a Lindberg tube furnace. The reaction was purged with argon at 200 cc/min for 20 min prior to heating. Gas flow was reduced to 100 cc/min and the furnace heated to 450 °C at a rate of 10 °C/min. The reaction was held at 450 °C for 60 min and then cooled to room temperature under argon. The black solid was cleaned in air using methanol to react excess NaBH_4_, HCl to convert residual sodium into sodium chloride and, finally, water to remove the sodium chloride. With each washing step, the solution was centrifuged at 10,000 rpm for ten minutes and the solvent removed. Severe aggregation of these ligand-free particles rendered electron-microscopy imaging infeasible. However, diffraction data suggest that the particles were approximately 17 nm, with the Scherrer equation giving calculated sizes of 17.46, 16.84, 17.62 and 17.21 nm, respectively, for x = 0.2, 0.5, 0.8 and 1.0.

Samples were analyzed by powder X-ray diffraction on a D8 Discover diffractometer (Bruker AXS Inc., Madison, WI, USA) operated at 35 kV and 40 mA using CoKα radiation. Samples were prepared for optical measurements by drop casting onto quartz slides. Raman spectra were collected on a LabRAM ARAMIS (HORIBA Jobin Yvon, Edison, NJ, USA) automated scanning confocal Raman microscope using a 532-nm excitation laser. Elemental analysis was performed by EDX spectroscopy on a Gemini Ultra-55 scanning electron microscope (Zeiss, Thornwood, NY, USA), and FTIR spectroscopy was performed on a Spectrum One equipped with an HATR assembly (PerkinElmer, Santa Clara, CA, USA). The absorbance was collected on a Cary-5000 UV-Vis-NIR (Agilent Technologies, Santa Clara, CA, USA). Samples were prepared for optical measurements by drop casting from water onto quartz slides, and the films were allowed to dry naturally in air. 

## 3. Results and Discussion

**Figure 1:**
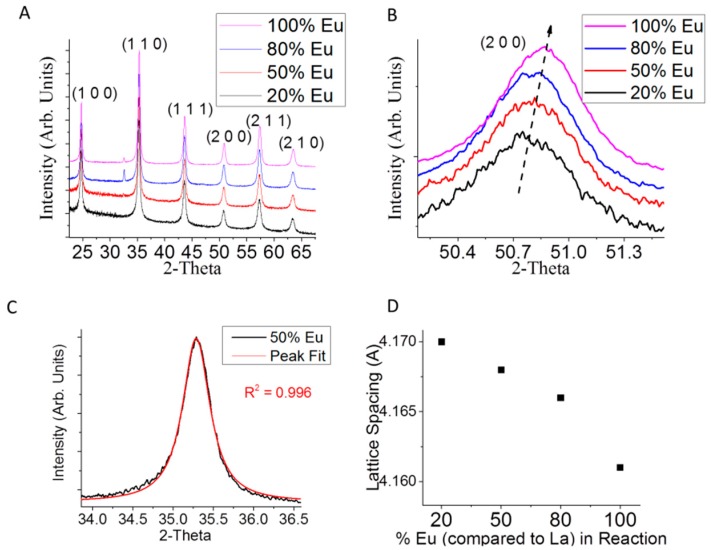
X-ray diffraction of (**A**) La_x_Eu_1−x_B_6_; (**B**) a magnified image of the (2 0 0) diffraction plane with La_x_Eu_1−x_B_6_ where x = 0.0, 0.2, 0.5 and 0.8; (**C**) Pearson VII peak fit of La_x_Eu_1−x_B_6_ with x = 0.5; and (**D**) lattice spacing versus atomic % Eu in the La_x_Eu_1−x_B_6_ reaction (calculated using Bragg′s law).

The success of the incorporation of a Eu into LaB_6_ was evident in changes to the XRD pattern of La_x_Eu_1-x_B_6_ (Figure 1A). Note that the small peak at ~33° is from an unidentified impurity in the EuCl_3_. Increasing the concentration of Eu in the La_x_Eu_1−x_B_6_ synthesis caused a shift of the diffraction pattern to higher 2-Theta (Figure 1B), which is indicative of increased compressive lattice strain. This seems counterintuitive since incorporating larger atoms typically expands a crystal lattice. For instance, in Eu_1-x_Ca_x_B_6_ the larger Eu atom replaces Ca and the lattice expands [21]. There is a possibility that increasing the amount of Eu in La_x_Eu_1-x_B_6_ may produce two phases, as reported for the (Ba_x_Ca_1−x_)B_6_ system which has a mixture of both Ba-rich and Ca-rich particles in the final product [22]. Though this could account for the unexpected change to the lattice strain in our system, the diffraction peaks of La_x_Eu_1−x_B_6_ are symmetric, which is indicative of a single phase (Figure 1C). In La_x_Eu_1-x_B_6_, there appears to be a decrease in lattice spacing with increasing Eu content (Figure 1D), even though Eu is larger than La. The B_6_ network, like all boron lattices, is electron-deficient and is only stable because of electron transfer from the metals [23]. Though Eu^2+^ and Ca^2+^ in Eu_1−x_Ca_x_B_6_ are different sizes they are also both divalent, so the free electron density does not change when increasing the Ca content. By contrast, in La_x_Eu_1−x_B_6_ there is a mix of trivalent La^3+^ and divalent Eu^2+^. This and the metal (M) vacancies within the system are likely responsible for the increasing lattice strain with increasing Eu concentration.

**Figure 2:**
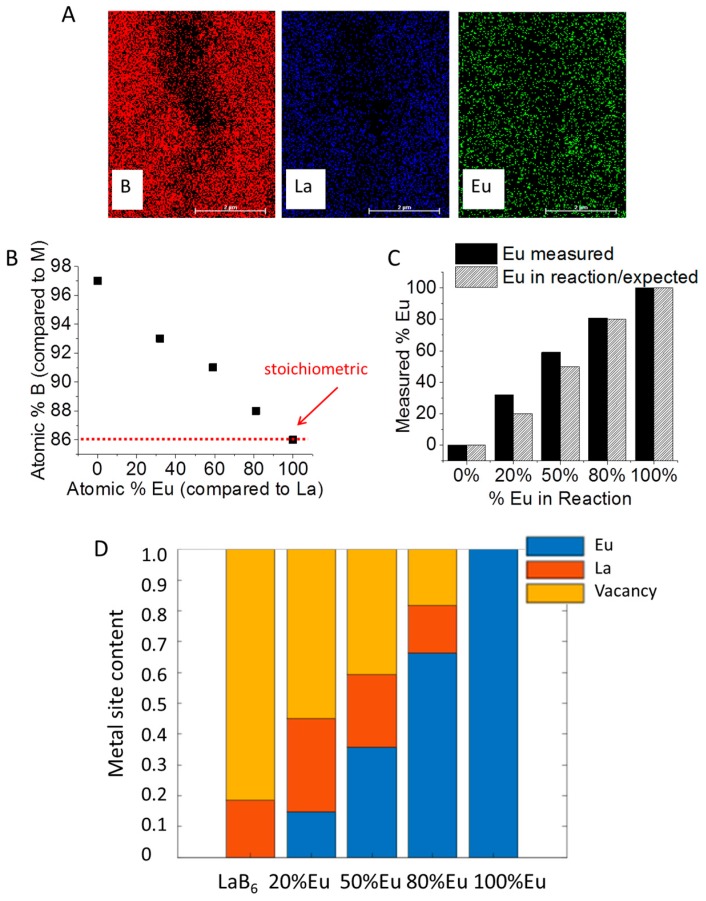
(**A**) View of the EDS map of La_x_Eu_1−x_B_6_ (x = 0.2) including B, La and Eu; (**B**) atomic % B versus atomic % Eu (the red dashed line is stoichiometric with 1M:6B); (**C**) measured versus expected % Eu (comparing Eu to La) in La_x_Eu_1−x_B_6_; and (**D**) metal content (Eu and La) and M void in La_x_Eu_1−x_B_6_.

EDS confirmed the presence of all three elements (La, B, and Eu) in La_x_Eu_1−x_B_6_ samples (Figure 2A; La_x_Eu_1−x_B_6_ with x = 0.2). Intriguingly, LaB_6_ synthesized under this method contained about 97% B, which indicates a huge amount of M vacancies with x = 0.19 (equivalent to about 80% M vacancies). M vacancies are common in LaB_6_, but it is understood that the lattice constant is unaffected by these voids [19,24,25]. he stability of the crystal structure is dictated by the bonds in the boron framework and not by the metal content so long as the electronic requirements of the structure are met [26]. However, if there is too much void space then MB_6_ becomes unstable. Though there is a lot of disagreement surrounding La-B phase diagrams, a B content above 90% [24,27] is expected to contain both LaB_6_ and an additional B phase [25,26,28,29], which suggests that any excess boron in our system may not lie within the MB_6_ structure. However, we see no indication of a separate B phase beyond La_x_Eu_1−x_B_6_ by XRD. The phase diagrams of La-B were developed under the assumption that high temperatures (≥1500 °C) are required to make LaB_6_, which was disproved only recently [15,30]. With low temperature reactions we recently reported the existence of bridging halogens between La atoms which are involved in the lattice formation of LaB_6_ [31,32,33], so even though a sample containing 97% B may potentially have a massive amount of vacancies, it′s possible that the structure was stable during formation because these halogens fulfilled the electronic requirements necessary to stabilize the material without the need of an additional B phase. Unfortunately, the amount of Cl in the materials reported here were either too low in concentration to be detected by EDS or the 450 °C reaction temperature was high enough to remove the bridging-Cl atoms as the final product formed. Work is ongoing understand exactly how halogen atoms enter into the reaction mechanism.

As the concentration of Eu in the La_x_Eu_1−x_B_6_ reaction is increased there is a clear trend of increasing amounts of B relative to M until the system becomes stoichiometric with EuB_6_ (86% B or x = 1; Figure 2B), with a slightly higher Eu content in La_x_Eu_1−x_B_6_ than was expected with x < 1 (Figure 2C). It′s possible that EuB_6_ is more energetically favored than LaB_6_ or that there are so many vacancies that at low concentrations the divalent Eu^2+^ has an easier time filling holes in addition to replacing La atoms. Regardless, there is a clear trend of decreasing vacancies with increasing Eu in the reaction (Figure 2D). Unfortunately, the ligand-free nature of these particles results in an aggregated product, rendering single-particle analysis on individual LaB_6_ particles infeasible.

**Figure 3:**
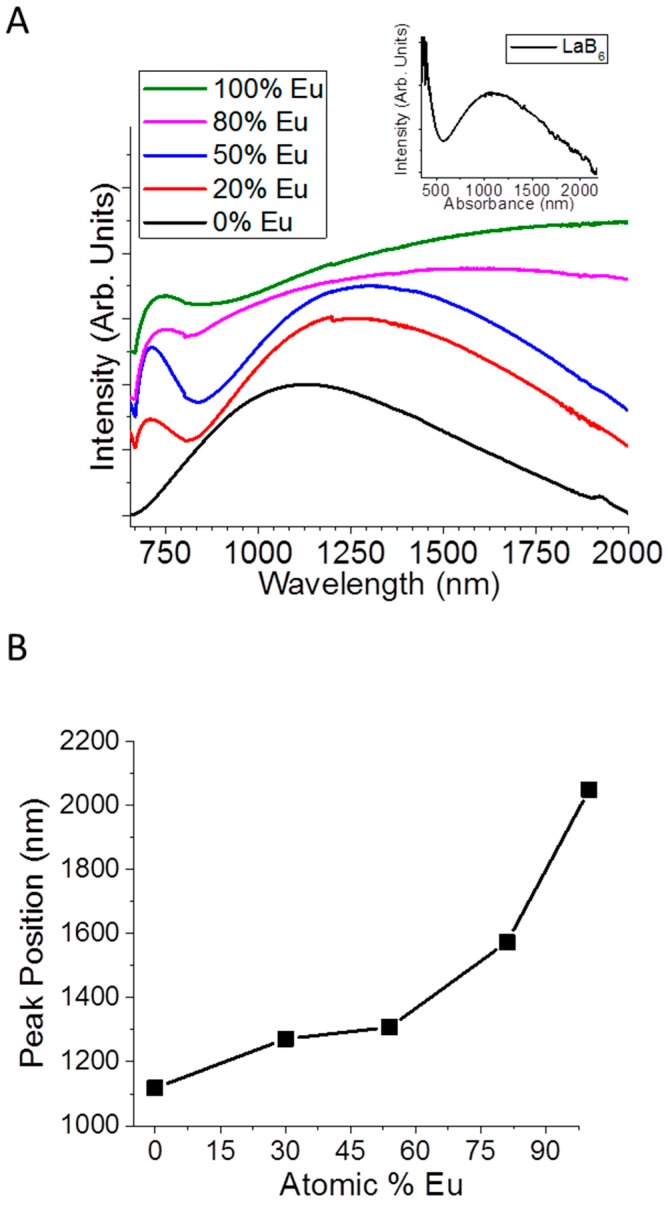
(**A**) Absorbance of La_x_Eu_1−x_B_6_ changing with Eu content (normalized) and (**B**) absorbance peak position versus atomic % Eu in La_x_Eu_1−x_B_6_.

There have been several publications studying the ability to tune the plasmon of LaB_6_ to achieve desired optical properties [13,14,15], but little is yet known about how vacancies influence these properties. Research discussing vacancies related to optical and vibrational properties in LaB_6_ are very recent [19,31], and though much has been done to study the magnetic and thermoelectric properties of La_x_Eu_1−x_B_6_ [34,35], no one until now has synthesized doped hexaborides at low temperatures. Furthermore, only in very recent years have the optical properties of doped MB_6_ been explored [36,37,38]. In this work, we used absorbance spectroscopy to determine how the Eu concentration and M vacancies in La_x_Eu_1−x_B_6_ nanocrystals can be used to tune the plasmonic properties (Figure 3A). When increasing the concentration of Eu the small absorbance peak in the visible region that is indicative of metal hexaborides shifts from ~380 nm in pure LaB_6_ to 730 nm in pure EuB_6_, while the larger absorbance peak red shifts from 1100 nm in pure LaB_6_ to 2050 nm in pure EuB_6_ (Figure 3B). Introducing Eu as a dopant causes a constant red shift of the absorbance peak from 1100 nm in pure LaB_6_ to 2050 nm in pure EuB_6_ (Figure 3B). This shift is a result of changes to the number of electrons in the conduction band as divalent Eu^2+^ replaces trivalent La^3+^. The sudden broadening of the absorbance at 80% Eu is most likely due to the changing carrier concentration which results from Eu incorporation as well as from changing metal vacancies within the lattice. 

**Figure 4:**
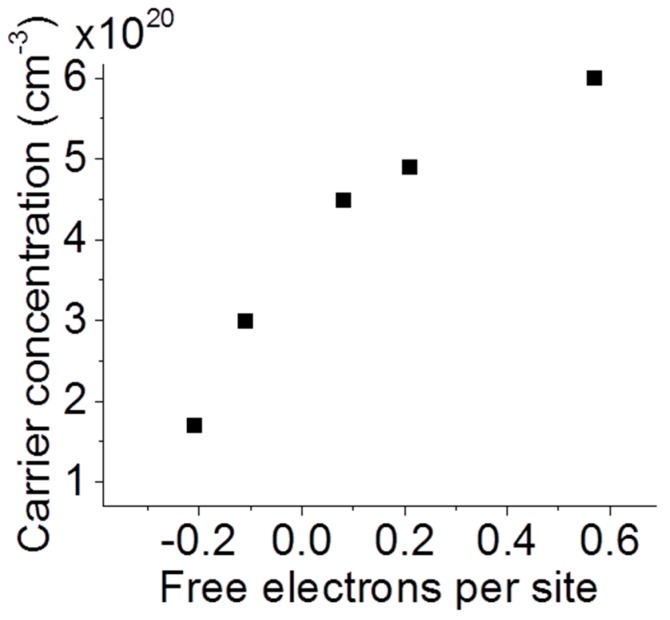
Localized surface plasmon resonance inferred carrier concentration versus number of free electrons per metal site in La_x_Eu_1−x_B_6_.

The electron deficiency is calculated as vacancy content minus lanthanum content. Whatever the mechanism causing the change in lattice spacing (vacancies or changing Eu content), the shifting absorption peak is indicative of an increase in carrier density with lanthanum content, and is impacted by vacancies within the system. Equation 1 gives the most basic model for the wavelength of the plasmon resonance [39],

(1) $$\lambda =2\pi c\sqrt{\frac{\varepsilon_{0}m*\left(\varepsilon \infty +k\varepsilon_{m}\right)}{Ne^{2}}},$$

where *N* is the number of charge carriers per unit volume, *e* is the charge of each carrier, m* is the effective mass of the charge carriers, *ε*_0_ is the permittivity of free space, *ε_m_* is the dielectric function of the surrounding medium, *ε_∞_* is the dielectric limit for the material at high frequencies (accounting for bound charge), and *k* is a geometrical factor. The absorbance spectroscopy was performed in air, so *ε_m_* is nearly unity. We treat the particles as spherical [15,19], which is associated with a constant of *k* = 2 and an effective electron mass of 0.225 *m*_0_ in EuB_6_ as reported based on optical measurements [40]. Finally, taking *ε_∞_* as unity, our absorption peaks translate to the charge concentrations in Figure 4. In short, Figure 4 illustrates qualitative agreement between increasing carrier concentration as inferred from plasmonic resonance and increasing carrier concentration as inferred from composition measurements. As the Eu content is increased the samples lose free electrons and the absorbance peak expands and broadens.

## 4. Conclusions

We have found that systematically increasing the amount of divalent Eu^2+^ compared to trivalent La^3+^ within Eu_1−x_La_x_B_6_ not only decreases the lattice spacing but drastically changes the vacancies within the system. These vacancies have a large influence on the optical properties and allow the plasmon to be tuned across an incredibly large range from 1100 nm to 2050 nm. The true nature of these particles on the nanoscale is not fully understood (i.e., the influence of Cl bridging atoms), but we are making great strides to improve our knowledge of this system. It is our hope that this work will not only help to further our understanding of the MB_6_ crystal structure, but may open new doors for developing new devices, optoelectronics, and more. Research is ongoing to study how this synthetic method may be used to alter the nanoparticle surface, bringing to light new properties which may become a vital aspect for biosensing applications.
